# Molecular cloning and characterization of *GhERF105*, a gene contributing to the regulation of gland formation in upland cotton (*Gossypium hirsutum* L.)

**DOI:** 10.1186/s12870-021-02846-5

**Published:** 2021-02-18

**Authors:** Chaofeng Wu, Hailiang Cheng, Shuyan Li, Dongyun Zuo, Zhongxu Lin, Youping Zhang, Limin Lv, Qiaolian Wang, Guoli Song

**Affiliations:** 1 Research Base, Anyang Institute of Technology, State Key Laboratory of Cotton Biology, Anyang, Henan 455000 China; 2 Institute of Cotton Research, Chinese Academy of Agricultural Sciences, Anyang, Henan 455000 China; 3 Huazhong Agricultural University, Wuhan, Hubei 430070 China; 4 Anyang Institute of Technology, Anyang, Henan 455000 China

**Keywords:** Cotton (*Gossypium hirsutum*, L.), *GhERF105*, Gland formation, Transcription factor

## Abstract

**Background:**

*Gossypium hirsutum* L. **(**cotton) is one of the most economically important crops in the world due to its significant source of fiber, feed, foodstuff, oil and biofuel products. However, the utilization of cottonseed was limited due to the presence of small and darkly pigmented glands that contain large amounts of gossypol, which is toxic to human beings and non-ruminant animals. To date, some progress has been made in the pigment gland formation, but the underlying molecular mechanism of its formation was still unclear.

**Results:**

In this study, we identified an AP2/ERF transcription factor named GhERF105 (GH_A12G2166), which was involved in the regulation of gland pigmentation by the comparative transcriptome analysis of the leaf of glanded and glandless plants. It encoded an ERF protein containing a converved AP2 domain which was localized in the nucleus with transcriptional activity, and showed the high expression in glanded cotton accessions that contained much gossypol. Virus-induced gene silencing (VIGS) against *GhERF105* caused the dramatic reduction in the number of glands and significantly lowered levels of gossypol in cotton leaves. *GhERF105* showed the patterns of spatiotemporal and inducible expression in the glanded plants.

**Conclusions:**

These results suggest that *GhERF105* contributes to the pigment gland formation and gossypol biosynthesis in partial organs of glanded plant. It also provides a potential molecular basis to generate ‘glandless-seed’ and ‘glanded-plant’ cotton cultivar.

**Supplementary Information:**

The online version contains supplementary material available at 10.1186/s12870-021-02846-5.

## Background

Cotton (*Gossypium* spp.) is a major crop worldwide for its economic value of the textile fiber, feed, foodstuff, oil and biofuel products in the world [1, 2]. There are approximately 50 species in the *Gossypium* genus, four of which are cultivated in agriculture, including two diploid cottons *(G.herbaceum* and *G.arboreum*) and two allotetraploid cottons (*G.hirsutum* and *G.barbadense*) [3–5]. among these species, the upland cotton, *G.hirsutum*, is most widely grown and dominates world cotton commerce with more than 90% of the annual cotton fiber production [5, 6]. However, the potential of nutrition sources of cottonseed cannot be fully realized due to the presence of gossypol glands in plants and seeds, which are toxic to human beings and non-ruminant animals [7, 8]. Gossypol, as phytoalexin, is a yellowish phenolic compound that serves as a protective function against various biotic and abiotic stresses in certain species of cotton plants of the family Malvaceae [9–11]. Therefore, developing cotton with low-gossypol seeds and high-gossypol plants has become an interesting area of cotton breeding for researchers.

The pigment glands, also called ‘gossypol glands’, ‘internal glands’ or ‘black glands’, located in the subepidermal layer of aerial organs in many parts of the plant, originate from a cluster of cells in the ground meristem, which differ from other cells in that they have a high-density gossypol and related terpenoids [7]. Research on the molecular genetic mechanisms of pigment gland in the cotton plant began in the lines of ‘Hopi Moencopi’ in the 1950s [12–14]. So far, many researches have indicated that the gland formation is controlled by a combination of at least six independent loci such as *gl*_*1*_, *gl*_*2*_, *gl*_*3*_, *gl*_*4*_, *gl*_*5*_ and *gl*_*6*_, the different combinations of dominant (*Gl*) and recessive (*gl*) alleles modulate gland formation in different organs [14–18]. The completely glandless phenotype was controlled by two pairs of duplicate homozygous recessive genes (*gl*_*2*_*gl*_*2*_*gl*_*3*_*gl*_*3*_) in the allotetraploid *G.hirsutum* [13, 14], while the dominant alleles *(Gl*_*2*_*Gl*_*2*_*,Gl*_*3*_*Gl*_*3*_) in any combination produced the glanded phenotype with variable distribution in different organs [14, 19]. The *gl*_*2*_ and *gl*_*3*_ genes were located on chromosome (chr.) A_t_12 and D_t_12 of *G.hirsutum*, respectively [16, 20, 21]. Alleles *gl*_*4*_ and *gl*_5_ decrease the number of glands while *gl*_*6*_ has the weaker effects on gland formation compared with *gl*_*1*_ [22, 23]. Subsequently, *gl*_*2*_^*arb*^, *gl*_*2*_^*b*^, *gl*_*3*_^*dav*^, *gl*_*3*_^*thur*^, *gl*_*3*_^*rai*^, *gl*_*3*_^*b*^ [7], *Gl*_*2*_^*s*^ [24], *Gl*_*2*_^*e*^ [25], *gl*_*3*_^*n*^ [26], and *Gl*_*2*_^*b*^ [27] related to pigment gland formation were also identified. Among them, *Gl*_*2*_^*e*^ is the most critical gene that controls glandless character of the whole plant. A single completely dominant glandless *G. barbadense* mutant (*Gl*_*2*_^*e*^) named ‘Bahtim 110’ (*G. barbadense* L), which is a dominant allele of *Gl*_*2*_ that shows epistatic effect on *Gl*_*3*_, was originally discovered in Egypt by the irradiation mutagenesis of the sea-island cotton ‘Giza 45’ seeds with ^32^P, and could efficiently inhibit the formation of pigment gland [28–31]. Since then, several genes for gland formation have been discovered gradually by researchers. In 2016, *GoPGF* gene (Gossypium Pigment Gland Formation gene), which encodes a basic helix-loop-helix transcription factor was identified through map-based cloning approach and located on chromosome A_t_12 [32, 33]. *CGF3* (Cotton Gland Formation), identical to *GoPGF* gene, not only controls the gland morphogenesis directly, but also regulates gossypol biosynthesis indirectly [34]. *CGP1* (Cotton Gland Pigmentation 1), which interacted with *GoPGF*, was identified by the comparative transcriptome analysis of glanded and glandless cotton accessions and involved in the regulation of gossypol biosynthesis but not gland formation [35]. In addition, the novel *RanBP2* zinc finger protein (ZFP) and *GauGRAS1*, which play the roles in the development of the cotton gland, were identified using suppression subtractive hybridization (SSH) from upland cotton ‘Xiangmian 18’ [9, 36–38]. during the past six decades, some progress has been made in the molecular mechanism of gland formation and the relationship between gossypol and pigment gland. However, the specific mechanism of pigment gland formation still remains unclear .

**Fig. 1:**
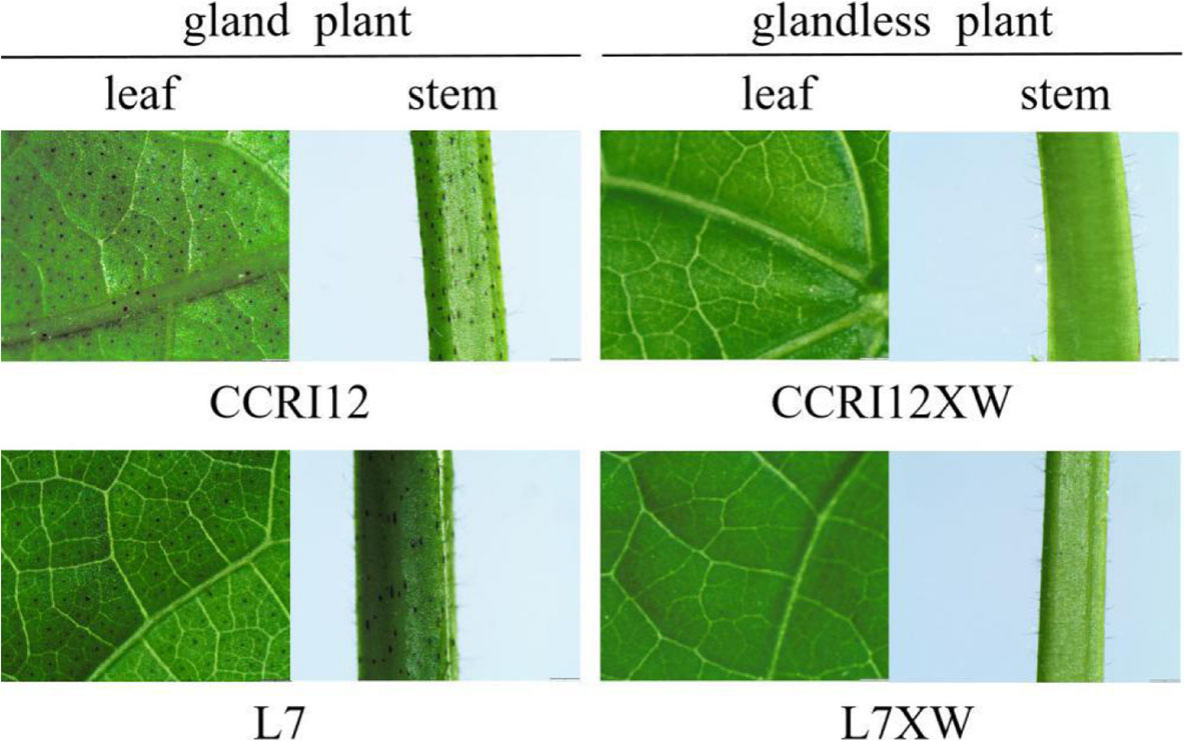
Gland traits of leaves and stems of glanded cultivar CCRI12 (or L7) and glandless cultivar CCRI12XW (or L7XW). CCRI12 (China Cotton Research Institute 12) and L7 (Liao Mian 7) are upland cotton cultivars with dark-colored pigment glands. CCRI12XW and L7XW are dominant glandless near-isogenic lines (NILs) that differ nearly only in the gland trait of CCRI12 and L7, respectively. Leaf and stem section from CCRI12 (or L7) showing presence of a typical array of glands, while Leaf and stem section from CCRI12XW (or L7XW) showing glandless. The images were taken by stereomicroscope (Olympus SZX10, Japan)

Here, we identified an Ethylene Response Factor named GhERF105, which was involved in the regulation of gland pigmentation, by the comparative transcriptome analysis of the leaf of two pairs of glanded and glandless cotton accessions, which are L7 and L7XW, CCRI12 and CCRI12XW(Fig. 1). The gene encoded an ERF protein localized in the nucleus with transcriptional activation activity containing a conversed AP2 domain and showed the high expression in glanded cotton accessions that contained much gossypol. Silencing of *GhERF105* by VIGS not only resulted in the drastic reduction of gland, but also decreased the accumulation of gossypol in the leaves of the treated plants. Moreover, *GhERF105* showed a temporal and spatial pattern of expression in various aerial organs of glanded and glandless cotton plants including cotyledon, hypocotyl, petiole, leaf and stem, and demonstrated the inducible expression under ethylene treatment. In addition, *GhERF105, CGF, CGP1* and *GoPGF* genes were highly expressed in the leaves and stems in glanded CCRI12 and L7 but had the lower expression in CCRI12XW, CCRI12YW and L7XW.

These results provide a reference for the comprehensive analysis of the molecular mechanism of gland formation and gossypol biosynthesis in cotton. However, the diversity of gland trait inheritance indicates the regulation complexity of gland formation. Further studies are needed to better understand the molecular mechanisms underlying gland development.

## Results

### Cloning and sequence analysis

In the study, 2009 DEGs between CCRI12 and CCRI12XW were identified, of which 1190 genes were downregulated (Supplementary Table S1), 980 DEGs between L7 and L7XW were identified, of which 541 genes were downregulated (Supplementary Table S2), 289 differentially co-expressed genes were obtained from the gland and glandless accessions, and represented down-regulated in the glandless accessions. Studies have shown that various transcription factors may be important for the formation of gossypol and the development of pigment glands [39–41]. Therefore, 14 transcription factors were identified from the 289 DEGs (Supplementary Table S3). The category of differentially expressed transcription factors genes encoded bHLH (GhMYC2-like) [32], followed by MYB (CGP1) [35], ERF (GhERF105), NAC and HSF. Programmed cell death (PCD) plays an important role during the development of pigment glands [42]. Evidence suggests ethylene were related to PCD by activating genes [43–45]. Therefore, we focused on an ethylene response factor. The *GhERF105* gene (GenBank ID: GH_A12G2166; accession number: XM_016865675), which was cloned from the leaves of CCRI12, is 711 bp in length containing an open reading frame (orf) with initial code (ATG) and terminal code (TAA) (Fig. S1). The predicted protein comprised of 236 amino acids with molecular mass of 26.3 kDa and isoelectric point of 7.72 containing an ERF conserved DNA binding domain (Fig.S2). The cotton GhERF105 belonged to the AP2/ERF family of transcription factors that play important roles in plant development and environmental stress responses, as well as hormone signaling and pathogen defense [46–48].

### The expression analysis of *GhERF105* gene in many cotton accessions

**Fig. 2:**
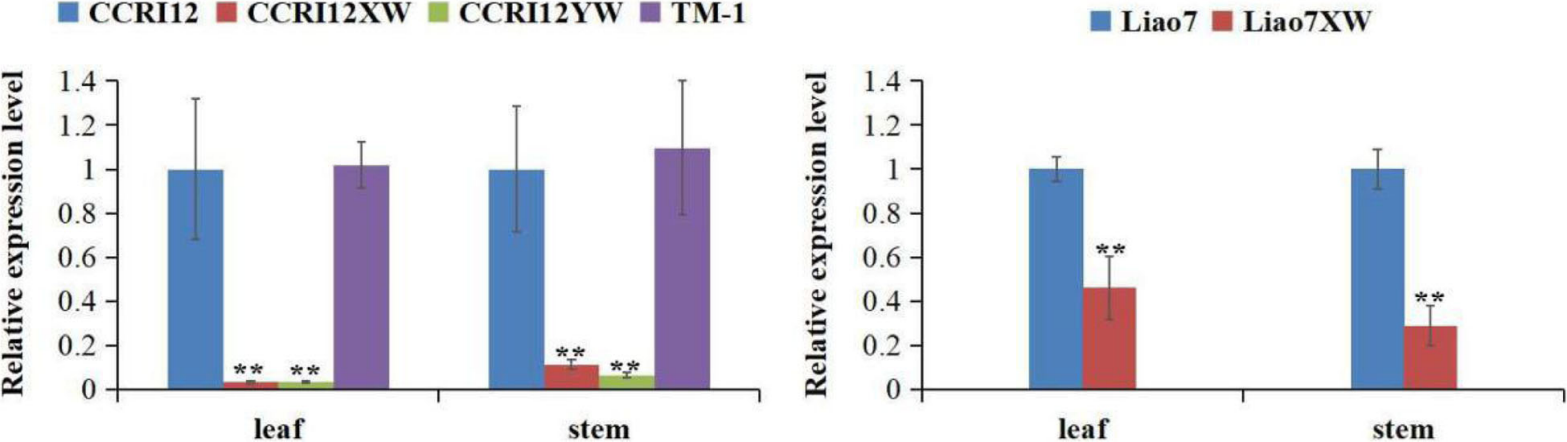
RT-qPCR expression analysis of *GhERF105* in the leaf and stem of CCRI12, CCRI12XW, CCRI12YW, L7, L7XW and TM-1. The experiment was performed using three biological replicates, statistical significance was analyzed using a Student’s t-tests (*P < 0.05, **P < 0.01). Error bars represent the standard of the mean values of three biological replicates.

The expression levels of *GhERF105* were analyzed in two pairs of Near Iso-genic Lines (NILs) and other cotton accessions,which showed that *GhERF105* was highly expressed in the leaves and stems of glanded *G.hirsutum.* (CCRI12, L7 and TM-1) but had indeed substantially lower expression in CCRI12XW, L7XW and CCRI12YW (Fig. 2). Based on the different expression pattern of *GhERF105* in partial organs of six cotton accessions, *GhERF105* may be related to the formation of glands. However, its function and regulatory mechanism in pigment gland development need further be investigated using VIGS technology and other technoloy.

### Silencing of *GhERF105* reduced gland formation and gossypol biosynthesis

**Fig. 3:**
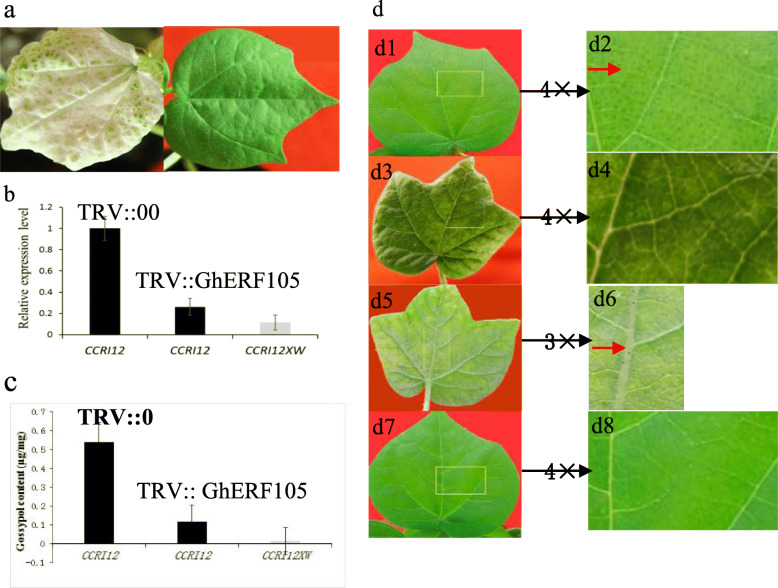
Functional characterization of *GhERF105* by VIGS. a The photo-bleaching phenotypes of cotton seedlings in CCRI12 inoculated with pTRV::GhPDS and empty vectors. TRV::GhPDS and TRV::00 are the positive control and negative control, respectively. b Relative transcript levels of GhERF105 of leaf inoculated with pTRV::GhERF105 or empty vector control. c The gossypol content in empty vector (TRV::00) and in the GhERF105-silenced leaves of CCRI12.and CCRI12XW, Actin was used as an internal control. d d1-d2 Phenotypes of *Gossypium hirsutum* CCRI12 inoculated with pTRV::00 vector. d3-d6 Phenotypes of *Gossypium hirsutum* CCRI12 inoculated with pTRV:: GhERF105 vector. d7-d8 Phenotypes of *Gossypium hirsutum* CCRI12XW. d1-d8 are enlarged versions of the positions indicated by the yellow box in Fig. 3. d1 correspond to d2, d3 correspond to d4, d5 correspond to d6 and d7 correspond to d8. the red arrow indicates the location of the glands on the leaf. Each bar value represents mean ± SD of three independent experiments

Here, in order to further ascertain the function of the *GhERF105* during pigment gland formation. *Agrobacterium*-mediated VIGS systems was constructed using a TRV-based VIGS vector for silencing phytoene desaturases gene (*GhPDS*) and *GhERF105* gene in the cotton seedlings. Results showed that silencing of PDS, caused loss of chlorophyll and carotenoids [49]. A photobleaching phenotype in cotton plants infiltrated with *GhPDS*-expressing agrobacteria was observed 14–21 days after infiltration in leaves, compared to the leaves in plants infiltrated with pTRV::00 agrobacteria (Fig. 3a). To assess its function, we cloned the 289 bp fragment of *GhERF105* from CCRI12 plant and inserted it into pTRV2 for virus-induced gene silencing (VIGS) to suppress the expression of endogenous in cultivated glanded allotetraploid cotton. Compared with that in the untreated CCRI12 as the negative con**t**rol (Fig. 3b1-b2), The *GhERF105*-silenced CCRI12 plants exhibited the dramatic reduction in gland numbers in the new leaf of 14-21d after infiltration (Fig. 3b3-b6). The transcript levels of *GhERF105* in pTRV-*GhERF10*5 leaves were prominently lower than those in the untreated CCRI12 but still higher than those in the untreated CCRI12XW (Fig. 3c). However, the veins of the new emerging leaves had fewer dotted glands and the stems had thickly dotted glands (Fig. 3b5-b6, Fig. S3). These data suggested that *GhERF105* regulated the glands formation in leaf but not stem, in contrast, *GoPGF* showed glandless phenotype in all organs including the leaves and stems [29]. We conducted HPLC analysis to measure the level of gossypol in the leaves, gossypol content was reduced by about 78% in the *GhERF105*-silenced leaves compared with the untreated CCRI12 leaves but still higher than those in the untreated CCRI12XW (Fig. 3d). In all, the results suggested that *GhERF105* might be involved in the pigment gland formation and gossypol biosynthesis.

### Spatiotemporal expression analysis of *GhERF105* gene

**Fig. 4:**
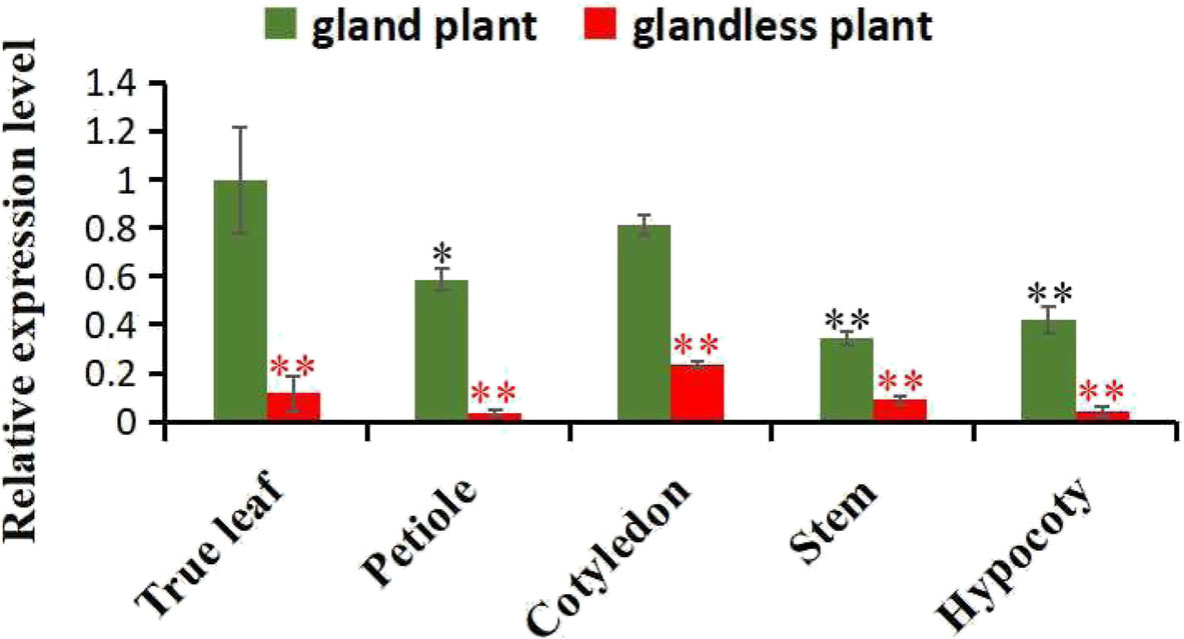
Spatiotemporal expression analysis of *GhERF105* gene in different organs of glanded plant and glandless plant. The experiment was performed using three biological replicates, statistical significance was analyzed using a Student’s t-tests (*P < 0.05, **P < 0.01). Error bars represent the standard of the mean values of three biological replicates. Red symbol ‘*’ indicates the expression of GhERF105 was significant difference in the same organ between glanded and glandless cultivars, Black symbol ‘*’ indicates the expression of GhERF105 was significant difference in the different tissues of glanded cultivars

The pigment glands are located on the surfaces of the stems, leaves, sepals, petals, and stigmas [17], *GhERF105* gene was associated with the development of cotton pigment gland. Therefore, the transcription level of *GhERF105* gene was detected by RT-qPCR in gland development of different organs of glanded and glandless cotton accessions. The result showed the mRNA levels in cotyledon, hypocotyl, petiole, leaf and stem of the gland plant were increased to 3.5, 10.5, 15.0, 8.7 and 4.0 folds of that in glandless plant, respectively. The mRNA levels of GhERF105 in the organs of the glanded plants were significantly higher than that in the glandless plants. At the same time, the expression level of GhERF105 was highest in the leaves of glanded plants but there wasn’t significant differences between the leaves and other organs of glandless plants (Fig. 4). In addition, there was no significant difference of GhERF105 between leaves and cotyledons of glandular cultivar, but significant difference from the petiole, hypocotyl and stem. Therefore, the *GhERF105* gene had highly different expression pattern between the glanded and glandless cotton plants in pigment gland formation.

### Nuclear localization and revealed transcription activity of GhERF105 protein

**Fig. 5:**
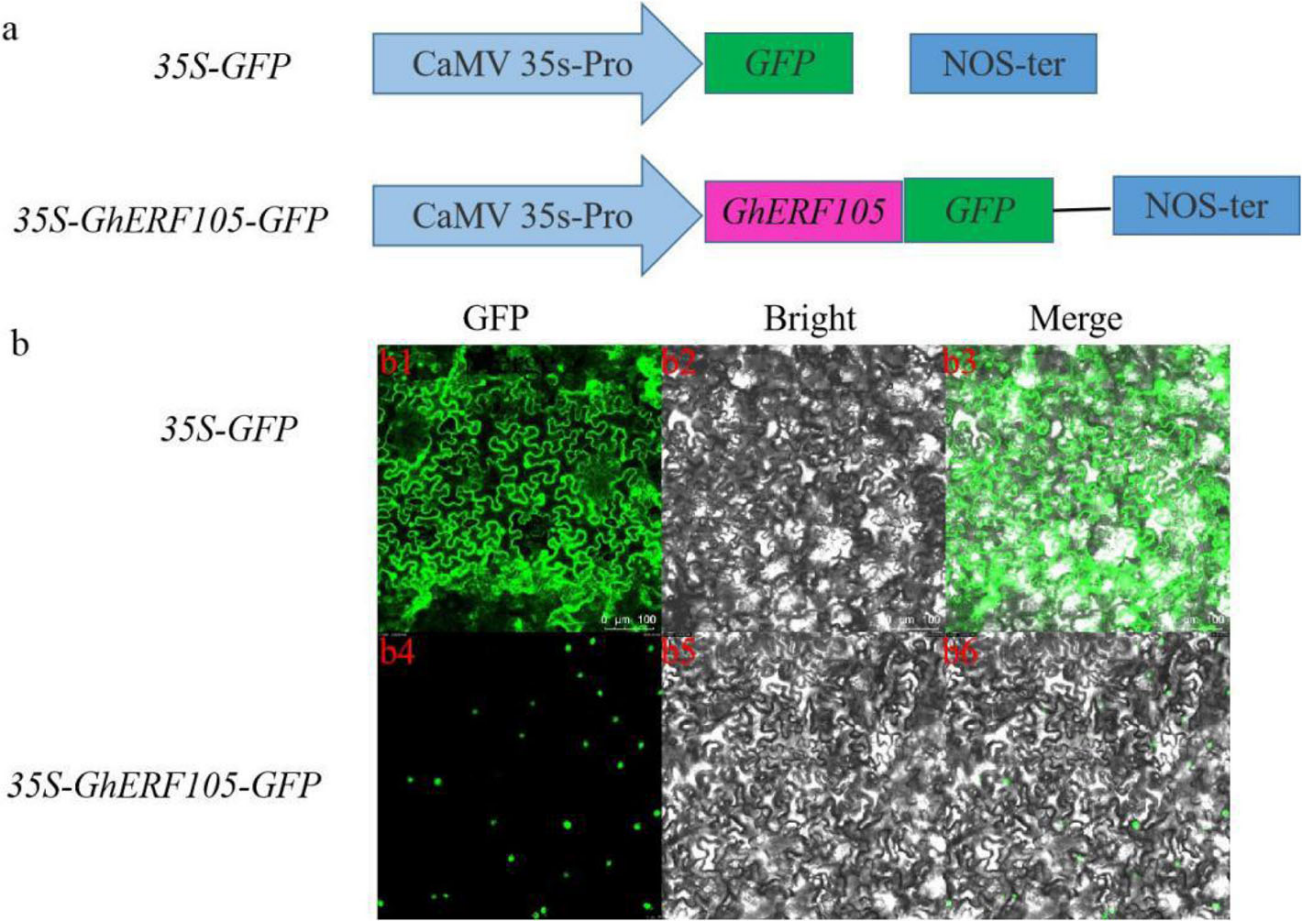
Subcellular localization of GhERF105. a The pBI121::GhERF105 fusion expression vector was constructed. b Subcellular localization of GFP (b1, b2 and b3) and GhERF105::GFP fusion protein (b4, b5 and b6) in the epidermal cells of leaves of *N. benthamiana*. Bar=100 μm

The green fluorescent protein (GFP) reporter, which is a vital marker for protein subcellular localization, showed a very strong fluorescence signal under the control of the constitutive CaMV35S promoter, and the signal was uniformly and diffusely distributed throughout the cell. Based on functional annotation information, GhERF105 is believed to act as a transcriptional factor. Therefore, the nuclear localization should be essential for the function of GhERF105. To test this hypothesis, the coding sequence (CDS) of *GhERF105* was fused to the green fluorescent protein (GFP) reporter gene. After introducing the construct (Fig. 5a, S4) into the tobacco cells by agro infiltration, GhERF105-GFP, the transcription factor fused to GFP, was expressed transiently and located exclusively in the nucleus of tobacco epidermal cells (Fig. 5b). The result confirmed that GhERF105-GFP was a nuclear localized protein.

**Fig. 6:**
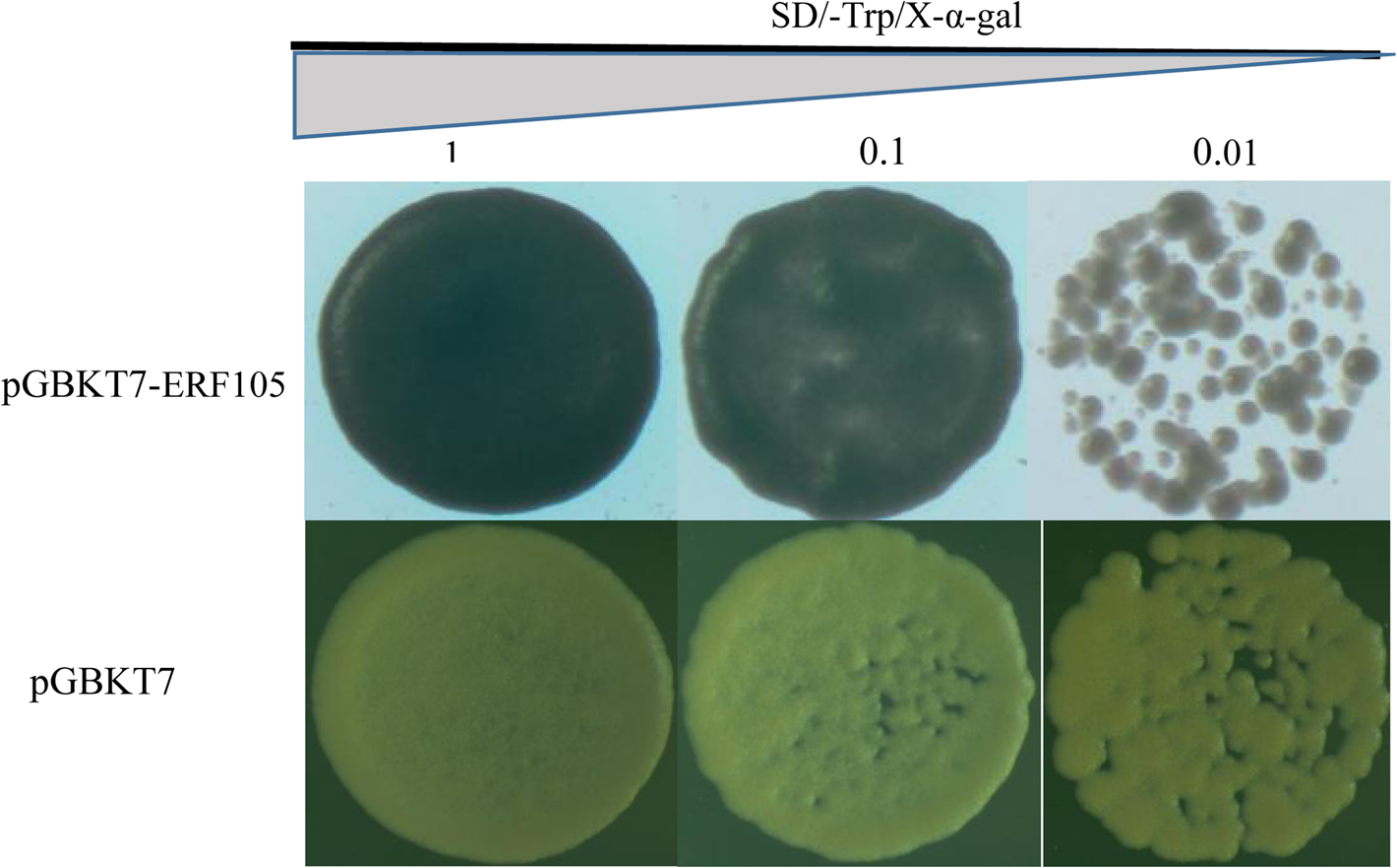
Transcription activation of GhERF105 in yeasts. The constructs pGBKT7 and pGBKT7 - GhERF105 were transformed in yeasts and grew for 3–4 days on the selected medium as indicated, Bar=200 μm

The yeast strains transformed with the pGBKT7-GhERF105 were able to grow blue colonies on the selective medium SD/−Trp/−X-a-gal while those strains with empty vector pGBKT7 could grow white colonies (Fig. 6). This result indicated that GhERF105 had the transcriptional activity, implicating a role of GhERF105 as a transcription activator.

### Expression pattern of *GhERF105* gene in cotton under ethylene treatment

**Fig. 7:**
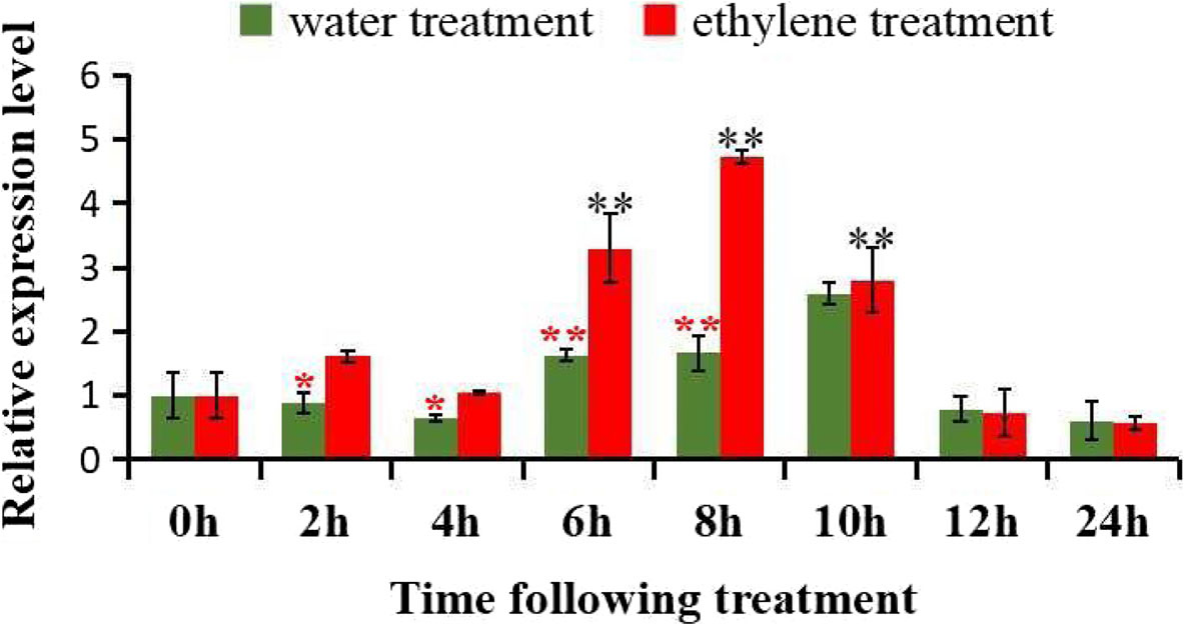
The expression of GhERF105 gene in leave of glanded plant under ethylene treatment. The samples were collected at 0, 2, 4, 6, 8, 10,12 and 24 h after ethylene treatment. The experiment was performed using three biological replicates, statistical significance was analyzed using a Student’s t-tests (*P < 0.05, **P < 0.01). Red symbol ‘*‘indicates the expression of GhERF105 was significant difference between ethylene treatment and water treatment at the same tissue, Black symbol ‘*‘indicates the expression of GhERF105 was significant difference at different time of ethylene treatment. Error bars represent the standard of the mean values of three biological replicates

The ERFs, which are important plant-specific transcription factors in the ethylene signal transduction pathway, have been shown to play a critical regulatory role in modulating the expression of specific stress-related genes [50–52]. Ethylene interact with other plant hormones and regulate the programmed expression of pathogenesis-related (PR) genes in the ethylene-mediated signaling pathways [53]. Programmed cell death (PCD) plays an important role during the development of pigment glands in *Gossypium hirsutum* leaf tissue [42]. Ethylene, which regulate the upstream signal molecular during PCD process, mediates the PCD signal by ROS [54]. Therefore, it is meaningful to investigate the expression pattern of *GhERF105* gene in response to stress hormone ethylene stimuli. In this study, RT-qPCR analysis was employed to detect the expression level of *GhERF105* in leaves at different times after ethylene treatment*.* Compared to that in the water-treated plants, the *GhERF105* mRNA was rapidly accumulated and reached the maximum at 8 h after ET treatment, followed by a rapid decline in 12–24 h and then declined to the original level in the ethylene-treated plants, These results suggested that the mRNA level of *GhERF105* gene was induced at the early stage of ethylene treatment and maintained the high level from 6 h to 10 h by the stress hormone ethylene in cotton leaves (Fig. 7**).** However, there wasn’t positive correlation between the expression change of GhERF105 and the length of time of ethylene treatment. These results indicated that the expression of *GhERF105* was responsive to ethylene treatment at the transcriptional level and *GhERF105* might be related to ethylene signal transduction pathways or defense/stress signaling pathways. At the same time, it is tempting to speculate that gland formation and gossypol synthesis in cotton might be induced and regulated directly or indirectly by ethylene.

### Expression patterns of genes involved in gland formation

**Fig. 8:**
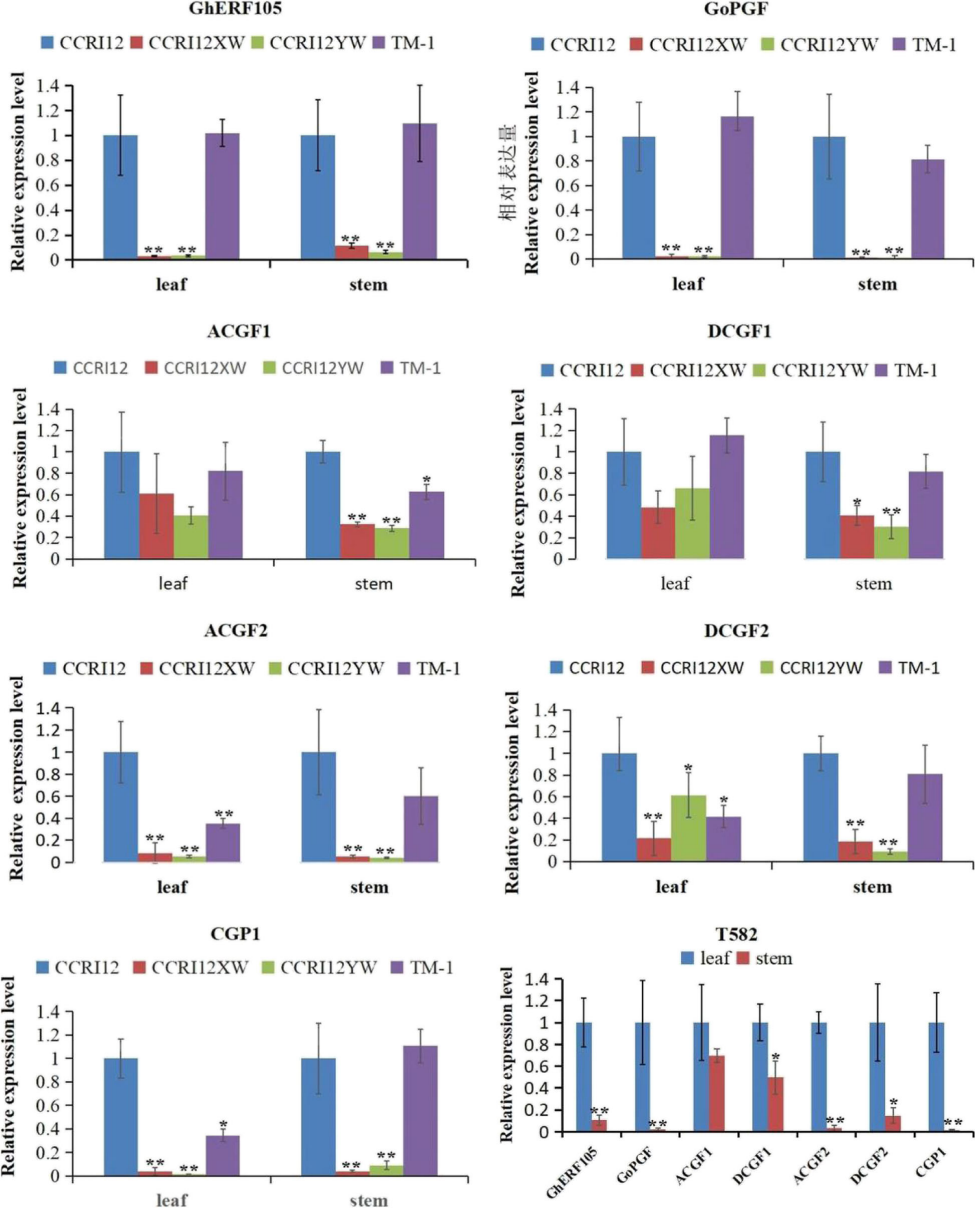
Expression analysis of *GhERF105, GoPGF, CGF1, CGF2* and *CGP1* genes in the leave and stem of glanded plant and glandless plant. The experiment was performed using three biological replicates, statistical significance was analyzed using a Student’s t-tests (*P < 0.05, **P < 0.01). Error bars represent the standard of the mean values of three biological replicates

*GoPGF*/*GhMYC2-like/CGF3* controled both gland morphogenesis and gossypol synthesis [33–35], *CGF1* showed similar functions to *GoPGF*/*GhMYC2-like*/*CGF3*, and *CGF2* regulated the density of pigment glands [35]. While *CGP1* regulated gossypol synthesis [36]. The expression levels of *GhERF105*, *CGF1*, *CGF2*, *CGP1* and *GoPGF*/*GhMYC2-like*/*CGF3* were analyzed by RT- qPCR in the leaf and stem of five cotton accessions including glanded *G. hirsutum* (CCRI12 and TM-1), dominant glandless CCRI12XW, recessive glandless CCRI12YW and glandless-stem and glanded-leaf accession (T582). Results obtained from RT- qPCR analysis confirmed that *GhERF105*, *CGF2*, *CGP1* and *GoPGF* were highly expressed in the leaves and stems in glanded CCRI12 and TM-1 but had lower expression in CCRI12XW and CCRI12YW. The expression of *CGF1* gene in the leaves of CCRI12, CCRI12XW and CCRI12YW was not significant, but it was significant difference in stems **(**Fig. 8**)**. In addition, we also observed that the expression level of these genes was significantly higher in the leaves than in the stems for *G. hirsutum (T582)* **(**Fig. 8**)**. These results showed that *GhERF105* had the similar expression pattern as *GoPGF*, *CGF1*, *CGF2* and *CGP1* in some cotton accessions. Conclusively, *GhERF105* was associated with cotton pigment gland development in leaves.

## Discussion

To date, developing cotton varieties, which produce low-gossypol seeds and high-gossypol plants, has become an important topic of cotton breeding. Therefore, it is very significant to understand the molecular mechanisms of the pigment gland formation and the relationship between gossypol and gland in cotton.

In the recent years, the considerable efforts have been made by researchers to accumulate knowledge and to identify a series of genes related to pigment gland formation and gossypol synthesis. *GoPGF/CGF3/GhMYC2-like* plays the critical role in gland development, independently regulates the gland morphogenesis and indirectly affects gossypol biosynthesis by regulating the expression of gossypol-related genes through binding to the G-box motif [34]. *CGF1* showed similar functions to *CGF3*, and *CGF2* regulates the density of pigment glands [35]. Silencing of *GoPGF* results in the absence of glands in all organs of glanded cotton and leads to an almost complete lack of gossypol [33–35]. Knockout of *CGP1* by CRISPR/Cas9 and VIGS produces a strong reduction in gossypol levels, showing that it modulates gossypol accumulation but not gland morphogenesis [36]. Silencing of *GauGRAS1* by VIGS leads to glandless stems and petiole and does not change the gland formation in the leaves in *G. australe*. Moreover, the gossypol content in the stem of the *GauGRAS1*-silenced plants was significantly reduced [38]. However, the molecular mechanism for pigment gland formation remains complicated and unclear, which leads to limit the progress in the breeding of low-gossypol cotton. Therefore, it is an intense need to explore the study on molecular mechanisms for gland formation which facilitate the genetic improvement of cotton.

This study provides several evidences that *GhERF105* gene was associated with gland formation in the partial organs of glanded plant. First, *GhERF105* gene was identified by the comparative transcriptome analysis of the leaf of glanded and glandless cotton accessions. Second, *GhERF105* was highly expressed in the glanded accession, while it had the lower expression in the glandless accession. Third, knockdown of *GhERF105* via VIGS markedly resulted in the drastic reduction of visible pigmented glands and decreased the content of the gossypol in the leaves but didn’t change the density of gland on the stem of the cotton. In addition, the expression pattern of *GhERF105* was similar with that of known genes related to gland development (such as *GoPGF* and *CGF*) in some glanded and glandless accessions (Fig. 8). These findings further indicated that *GhERF105* might be involved in the gland formation in cotton. Nevertheless, the regulatory mechanism on the pigment gland was somewhat different between *GhERF105* and *GauGRAS1*.

**Fig. 9:**
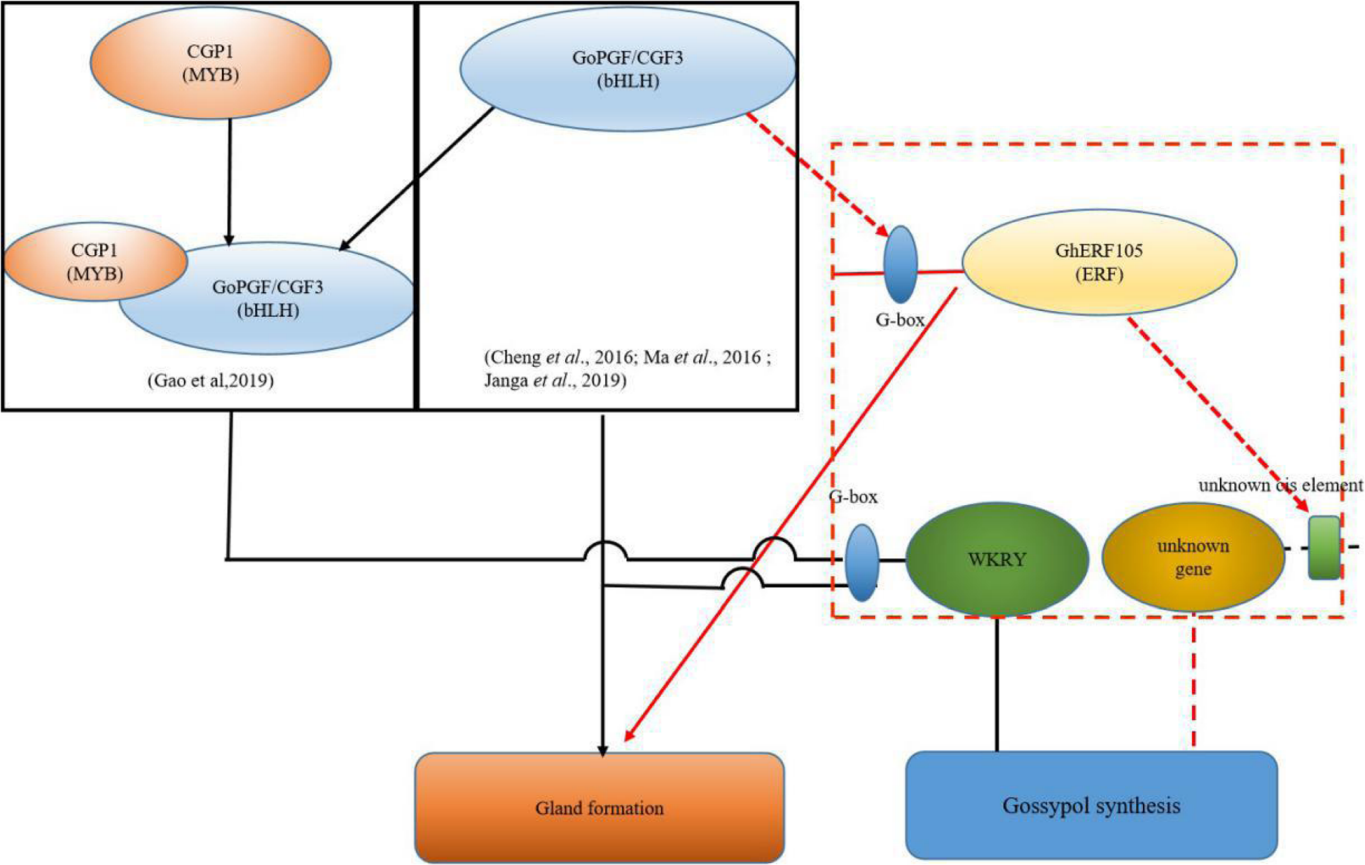
Schematic model illustrating the proposed functions of GhERF105 and in cotton gland pigmentation formation. Black parts are confirmed, red parts are suggested in the current study

Gao et al.(2019) had proved that CGP1a interacts with GoPGF in the tobacco cell nucleus, regulates multiple gossypol biosynthetic genes and controls gossypol and other terpenoid compounds [36]. Ma et al. (2016) had confirmed that *GoPGF* independently regulates gland morphogenesis and gossypol synthesis by binding the G-box motif present in the promoters of WRKYs and terpene synthases (TPSs) respectively by Yeast one-hybrid assays [34]. The promoter region of *GhERF105* includes G-box cis-acting elements. It is speculated that *GoPGF* regulates the expression of *GhERF105* by binding to G-box cis-acting elements in the nucleus and modulates the expression of gossypol-related genes by binding to the related cis-acting elements of their promoter directly and indirectly (Fig. 9). This speculation will be needed to be further verified by the results of related experiments.

In conclusion, the cloning and characterization of *GhERF105* both provide new information to study the molecular mechanism of gland formation and its functions in upland cotton.

## Conclusions

Based on the comparative transcriptome analysis of the leaf from two pairs of glanded and glandless cotton plants, we identified an ethylene response factor named *GhERF105* that was involved in the regulation of gland pigmentation, The *GhERF105* gene, which was cloned from the leaves of CCRI12, had 711 bp in length containing an open reading frame (orf) with initial code (ATG) and terminal code (TAA), The predicted protein comprised of 236 amino acids with relative molecular weight of 26.3 kDa and isoelectric point of 7.72 containing an ERF conserved DNA binding domain. The cotton GhERF105 belonged to the largest AP2/ERF family of regulatory transcription factors. The gene was differentially expressed in different organs from glanded and glandless cotton accessions. Silencing of *GhERF105* by VIGS not only reduced the number of glands, but also decreased the accumulation of gossypol in the leaves of treated plants. GhERF105 was located in the nucleus with transcriptional activation activity and induced by ethylene. The results suggested that the novel *GhERF105* may contribute to the regulation of the pigment gland and gossypol biosynthesis, as well as hormone signaling and pathogen defense.

Taken together, the cloning and characterization of *GhERF105* gene will open novel opportunities to discover the molecular mechanism of gland formation in cotton. These results will further facilitate the improvement of cotton varieties with glandless seeds and glanded plants through genetic engineering.

## Methods

### Plant materials and growth conditions

Accessions of CCRI12, CCRI12XW, CCRI12YW, L7, L7XW, TM-1, T582, were obtained from Cotton Research Institute, the Chinese Academy of Agricultural Sciences (CAAS) (Anyang, China). Among these, CCRI12 (China Cotton Research Institute 12) and L7 (LiaoMian 7) are upland cotton cultivars with dark-colored pigment glands and high content of gossypol in both plants and seeds. While CCRI12XW, CCRI12YW, and L7XW, which have glandless and low gossypol content in both seeds and plants, are dominant glandless near isogenic lines (NILs) that differ primarily in the gland trait of CCRI12 and L7, respectively [55]. ‘TM-1’, which is widely used as a genetic standard, is the glanded accession of the seeds and the whole plant, ‘T582’ is an accession with glandless-stem and glanded-leaf of plant. All materials were maintained by self-crossing for several years in our lab.

The seeds were immersed in water and followed by germination in a high humidity environment at 28 °C in the dark for 2 d. Well-germinated seeds were subsequently planted in 0.3 L pots of 7 cm diameter with one seed per pot in a commercially available sand/soil/fertilizer mix and grown for two to 3 weeks at 28 °C (16 h light and 8 h dark) with LED lamps (Opple lighting Zhongshan China) in a greenhouse.

### Extraction of total RNAs and synthesis of cDNA

Samples from different organs of the cotton plants, including cotyledon, hypocotyl, petiole, leaf and stem of one or many different gland accessions, served as the source of total RNA, were immediately frozen in liquid nitrogen and stored at − 80 °C. Total RNAs were isolated from 100 mg of leaf ground with liquid nitrogen using the RNAprep Plant RNA kit (polysaccharides&polyphenolics-rich) (TIANGEN BIOTECH (BEIJING)CO., LTD) according to the manufacturer’s instructions. The quantity and purity of RNAs were assessed according an absorbance ratio of OD _260/280_ (1.9–2.1) using a NanoDrop One^C^ Microvolume UV-Vis Spectrophotometer with Wi-Fi (Thermo Fisher Scientific Inc., Waltham, MA, USA) ultraviolet spectrophotometer, and was confirmed using 1.0% (w/v) denatured formaldehyde agarose gel electrophoresis to investigate its integrality. First strand cDNA was synthesized by the PrimmeScript™ II 1st strand cDNA Synthesis Kit (TaKaRa Bio, Dalian, China) following the manufacturer’s protocol of Reverse Transcription System.

### RNA-sequencing

Near-isogenic lines of tetraploid cotton (*Gossypium hirsutum* L.) cultivars CCRI12, L7 and glandless lines CCRI12XW, L7XW were used for comparative RNA-seq analysis to identify the genes that are involved in gland formation. Leaves of each lines were collected for library preparation and RNA-sequencing were performed using Illumina HiSeq 2000. DESeq2 program was used to identify differentially expressed genes, (log fold change ≥1 and FDR< 0.05) were considered to be the cutoff threshold to determine differentially expressed genes [56–59]. All sequencing data have been deposited in SRA (www.ncbi.nlm.nih.gov/sra). The accession numbers are SRR1652340, SRR1652393, SRR1652399 and SRR1652403.

### Molecular cloning of *GhERF105* gene

The full-length cDNA sequence of *GhERF105* was amplified from the leaves of CCRI12, and cloned into the pBI121 vector for sequencing (Sangon, Shanghai, China or Genewiz, Suzhou, China). PrimeSTAR®GXL DNA polymerase, dNTPs and other reagents were supplied by TaKaRa Bio, (Dalian) Co., Ltd. PCR amplification of the *GhERF105* gene was performed in a reaction volume of 15 μL containing 1 μL of template cDNA, 1.2 μL of dNTP Mixture (2.5 mM), 3 μL of 5×PrimeSTAR GXL Buffer, 0.3 μL of PrimeSTAR® GXL DNA Polymerase, 0.4 μL of forward primer and 0.4 μL of reverse primer (10 μΜ for each), and 8.7 μL ddH_2_O. The reaction procedure: 5 min at 98 °C; 35 cycles of 10 s at 98 °C, 15 s at 55 °C, 1 min at 68 °C and a final cycle of 5 min at 68 °C; hold at 10 °C. The PCR products were then purified following the instructions in the QIAquick PCR Purification Kit (250) (Qiagen, Düsseldorf, Germany) and eluted in a final sample volume of 35 μL of Qiagen EB buffer. The primers used for *GhERF105* cloning were listed in Supplementary Table S4.

### Identification of GhERF105 gene and its promoter in *Gossypium hirsutum*

The amino acid sequences and AP2 domain of GhERF105 were obtained from CottonFGD (https://cottonfgd.org/about/download.html) and Prosite (https://prosite.expasy.org/)respectively, The predicted molecular weight and isoelectric points of GhERF105 protein were calculated using the ExPASy program (http://web.expasy.org/protparam/). The G-box of the promoter of GhERF105 were obtained from PLANTCARE (http://bioinformatics.psb.ugent.be/webtools/plantcare/html/).

### Gene expression analysis by real-time quantitative PCR

Expression levels of *GhERF105* was performed by real-time quantitative (RT-qPCR) analysis using the ABI Quantstudio 5 Detection System (Applied Biosystems, Carlsbad, CA). Actin (GenBank accession numbers: AY305733) was used as reference gene. The 20 μL RT- qPCR experiment was carried out with TB Green Premix Ex Taq™ (Tli RNaseH Plus) (TaKaRa Bio, Dalian, China). The reaction contains 0.5 μL of each primer (10 μM), 0.4 μL ROX Reference DyeII (50x), 1 μL above synthesized cDNA template, and 7.6 μL of sterilized ddH_2_O. The conditions were as follows: one cycle at 95 °C for 5 min, 40 cycles of 95 °C for 5 s, 55 °C for 30 s, and 72 °C for 30 s. Each sample was run in triplicate, each biological replicate was assessed three times. The relative expression level of the genes was calculated according to the 2^−ΔΔCT^ method [59]. The primers were designed using the Primer 5.0 software or online in NCBI website (https://www.ncbi.nlm.nih.gov/tools/primer-blast/index.cgi? LINK_LOC=BlastHome) and listed in Supplementary Table S5.

### VIGS procedure

For knockdown of *GhERF105* gene, The pTRV-VIGS vectors were constructed using a previously published method [60–63]. Briefly, cDNA fragments of cotton PDS (*GhPDS1*, 327 bp, GenBank accession numbers: HQ441184) and Pigment gland formation *GhERF105* (337 bp) were amplified using Prime STAR GXL DNA Polymerase (TaKaRa) from CCRI12 by PCR with gene-specific primers (listed in the Supplementary Table S6). The resulting products were cloned into pTRV2 with *BamHI* and *KpnI* to produce recombinant vectors named pTRV2::PDS and pTRV2::GhERF105, respectively. These recombinant vectors and the empty vector (pTRV2::00) were then introduced into the *Agrobacterium* strain GV3101 (Weidi Bio, Shanghai, China) by heat shock method. Agrobacterium cultures containing pTRV1 and pTRV2 or its derivatives (pTRV2::PDS and pTRV2::GhERF105) were mixed in a 1:1 ratio. Seedlings with the fully expanded cotyledons but without a visible leaf of CCRI12 were infiltrated by inserting the *Agrobacterium* suspension containing pTRV1 and pTRV2, pTRV2-GhPDS, pTRV2-GhERF105 into the cotyledons via a syringe respectively. Plants were grown at 25 °C with a 16 h light / 8 h dark photoperiod with 70% humidity [33]. To analyze silencing efficiency, RNA was extracted and RT-qPCR was performed. The *Actin* (GenBank accession numbers: AY305733) and *GhERF105* was amplified as reference gene and target gene, respectively [64]. In this study, leaves 2–3 were investigated and collectively referred to as total foliage [65]. All primers used in this experiment were listed in Supplementary Table S6.

### Gossypol content and analysis

The gossypol was extracted from the leaves of CCRI12, *GhERF105*-silenced CCRI12 and CCRI12XW plants by high-performance liquid chromatography (HPLC) (Agilent 1100, Agilent, Santa Clara USA) [33, 66]. Each 100 mg plant sample were freeze-dried and ground into powder using liquid nitrogen, the 2 ml leaf extraction (acetonitrile/water/phosphoric acid=80:20:0.1) was added. The extraction was centrifuged at 10000 rpm for 10 min and then the supernatant was carefully transferred into a new EP tube. The eluent was filtered using a 0.45 μm nylon filter into a vial. The extract was analyzed using HPLC. A gossypol reference standard was purchased from Sigma Chemical Co. Ltd.

### Subcellular localization of GhERF105 protein

To study the subcellular localization of GhERF105 protein, the coding regions of GhERF105 was amplified with stop codon removed by Primers listed in Supplementary Table S7, which contained a *XbaI* and *SmaI* site (underlined) through polymerase chain reaction (PCR), The resulting fragments were cloned between the *XbaI* and *SmaI* site of the transient expression pBI121-GFP vector, which harbors an ORF encoding the green fluorescent protein (GFP) under the control of the CaMV35S promoter, and construct the recombinant plasmid *p35S*-*GhERF105*-*GFP*. p35S-GFP was used as positive control. The plasmids of GFP-GhERF105 and GFP were then introduced into tobacco leaves (*Nicotiana benthamiana*) respectively via *Agrobacterium*-mediated transformation and incubated at 25 °C under light for 48–72 h. The green fluorescence signals were observed and the localization of the fusion protein was determined using a confocal laser scanning microscope (Leica TCS SP8, Germany).

### Transactivation activity assay of GhERF105 protein

To study the transactivation activity of GhERF105 protein, GhERF105 cDNA was amplified with Primers listed in Supplementary Table S8 and cloned into the *EcoRI and NotI* sites of pGBKT7 vector to generate pGBKT7-GhERF105 construct. This plasmid with empty vector control was then transformed into yeast strain AH109 to analyze the transactivation activity. Yeast transformants with OD600 of 0.1, 0.01and 0.001 were plated on the selective media, SD/−Trp and SD/−Trp/−X-a-gal, and incubated at 30 °C for 4 d.

### Ethylene treatment

Ethephon (ET), which emits ethylene when dissolved in water, was used as a substitute for ethylene. Leaves from normally grown 3-to 4-week-old plants were used during the trefoil stage. Compared to the leaves sprayed with the water as negative control, Ethylene treatment was performed by spraying the leaves with the mixture of 1 mM/L ethephon (Solarbio Bio, Beijing, China). before leaf tissue was sampled, All the control and treated plants were enclosed in plastic bags for different time and place in a sealed chamber at 25°Cwith a 16-h-light/8-h-dark photoperiod. The whole plants were harvested at 0, 2, 4, 6, 8, 10, 12 and 24 h after treatments. Immediately frozen in liquid nitrogen and stored frozen at − 80 °C until use. The primers used for expression analysis were listed in Supplementary Table S5.

### Statistical analyses

All experiments were performed at least three times, and the results represent the mean ± standard deviation (SD) of three replicates. Statistical significance of the data was evaluated using one-way ANOVA using GraphPad Prism 8.0 or the SPSS software (version 22.0). A *P-*value < 0.05 was considered significant. A *P-*value < 0.01 was considered highly significant.

## Supplementary Information

**Additional file 1 Table S1** The downregulated DEGs between Z12 and Z12XW. **Table S2** The downregulated DEGs between L7 and L7XW. **Table S3** Categories of transcription factors in DEGs. **Table S4** List of primers used for cloning GhERF105. **Table S5** Primer of Real-time PCR used in the expression analysis of GhERF105 and other genes. T**able S6** List of primers used in VIGS experiment. **Table S7** List of primers used in subcellular localization. **Table S8** List of primers used in transactivation activity experiment.

**Additional file 2 Fig. S1** Amplification of the full-length cDNA of *GhERF105*. 1: DNA marker; 2–3: the full-length cDNA of *GhERF105*. **Fig. S2** Nucleotide and amino acid sequences of *GhERF105*. Symbol ‘*’ indicates the amino acid encoded by TGA, The conserved ERF domain is in black bold. **Fig. S3** The phenotypes of stem inoculated with pTRV::GhERF105 and empty vector (pTRV::00)**. Fig. S4** The double digestion by *XbaI* and *SmaI* of result of pBI121-GhERF105-GFP construction. 1: DNA marker (DM15000), 2 double digestion by endonuclease of recombinant vector. 3 DNA marker (DM2000).

## Data Availability

Extra data has been appended as supplementary Tables. All sequencing data have been deposited in SRA (www.ncbi.nlm.nih.gov/sra). The accession numbers are SRR1652340, SRR1652393, SRR1652399 and SRR1652403 available at https://www.ncbi.nlm.nih.gov/sra/.

## Abbreviations

CDS: The coding sequence
DEGs: Differentially expressed genes
ERF: Ethylene responsive factor
ET: Ethephon
FDR: False discovery rate
GFP: Green fluorescent protein
ORF: Open reading frame
PCD: Programmed cell death
VIGS: Virus induced gene silencing

## References

[1] Sunilkumar G, Campbell LM, Puckhaber L, Stipanovic RD, Rathore KS (2006). Engineering cottonseed for use in human nutrition by tissue-specific reduction of toxic gossypol. Proc Natl Acad Sci USA.

[2] Wang K, Wang Z, Li F, Ye W, Wang J, Song G (2012). The draft genome of a diploid cotton Gossypium raimondii. Nat Genet.

[3] Bao Y, Hu G, Flagel LE, Salmon A, Bezanilla M, Paterson AH (2011). Parallel up-regulation of the profilin gene family following independent domestication of diploid and allopolyploid cotton (Gossypium). Proc Natl Acad Sci USA.

[4] Wendel JF (2000). Genome evolution in polyploids. Plant Mol Biol.

[5] Zhang HB, Li YN, Wang BH, Chee PW. Recent advances in cotton genomics. Int J Plant Genomics. 2008;2008:742304. 10.1155/2008/742304. 10.1155/2008/742304 PMC2233810 18288253

[6] Yu J, Kohel RJ, Smith CW (2010). The construction of a tetraploid cotton genome wide comprehensive reference map. Genomics.

[7] Bell AA. and Stipanovic RD. The chemical composition, biological activity, and genetics of pigment glands in cotton. In: Proceedings of the Beltwide Cotton Conferences, 10-12 January 1977, Atlanta, GA. Memphis: Cotton Foundation Publisher; 1997. p. 244-258.

[8] Zhang WJ, Xu ZR, Pan XL, Yan XH, Wang YB (2007). Advances in gossypol toxicity and processing effects of whole cottonseed in dairy cows feeding. Livest Sci.

[9] Cai YF, Xie YF, Liu JG (2010). Glandless seed and glanded plant research in cotton. A review. Agron Sustain Dev.

[10] Gao W, Long L, Zhu LF, Xu L, Gao WH, Sun LQ (2013). Proteomic and virus-induced gene silencing (VIGS) Analyses reveal that gossypol, brassinosteroids, and jasmonic acid contribute to the resistance of cotton to Verticillium dahliae. Mol Cell Proteomics.

[11] Tian X, Ruan JX, Huang JQ, Yang CQ, Fang X, Chen ZW (2018). Characterization of gossypol biosynthetic pathway. Proc Natl Acad Sci USA.

[12] McMichael SC (1954). Glandless boll in upland cotton and its use in the study of natural crossing. Agronomy J.

[13] McMichael SC (1959). Hopi Cotton, a Source of Cottonseed Free of Gossypol Pigments 1. Agronomy J.

[14] McMichael SC (1960). Combined effects of glandless genes gl2 and gl3 on pigment glands in the cotton plant. Agronomy J.

[15] Gutierrez M, Vrdoljak J, Ricciardi A (1972). Development of gossypol-glandless strains of cotton. In Induced Mutations and Plant Improvement.

[16] Lee JA (1965). The genomic allocation of the principal foliar-gland loci in Gossypium hirsutum and Gossypium barbadense. Evolution.

[17] McCarty JC, Hedin PA, Stipanovic RD (1996). Cotton Gossypium spp. plant gossypol contents of selected GL_2_ and GL_3_ alleles. J Agric Food Chem.

[18] Scheffler JA, Romano GB (2012). Registration of GVS1, GVS2, and GVS3 upland cotton lines with varying gland densities and two near-isogenic lines, GVS4 and GVS5. J Plant Reg.

[19] Miravalle RJ, Hyer AH (1962). Identification of the Gl2 gl2 Gl3gl3 genotype in breeding for glandless cottonseed. Crop Sci.

[20] Endrizi JE, Turcotte EL, Kohel RJ (1985). Genetics, cytology, and evolution of Gossypium. Adv Genet.

[21] Percy R, Hendon B, Bechere E, Auld D, Fang DD, Percy RG (2015). Qualitative genetics and utilization of mutants. Cotton.

[22] Lee JA (1962). Genetical studies concerning the distribution of pigment glands in the cotyledens and leaves of upland cotton. Genetics.

[23] Lusas EW, Jividen GM (1987). Glandless cottonseed: a review of the first 25 years of processing and utilization research. J Am Oil Chem Soc.

[24] Barrow JR, Davis DD (1974). Gl_2_^s^-a new allele for pigment glands in cotton. Crop Sci.

[25] Kohel RJ, Lee JA (1984). Genetic analysis of Egyptian glandless cotton. Crop Sci.

[26] Zhang TZ, Zhang XL, Jin L, Chen ZX, Guo WZ (2001). Genetic identification of a new gland forming gene in upland cotton. Acta Agron Sin.

[27] Zhu SJ, Reddy N, Jiang YR, Ji DF (2004). Breeding, introgression and inheritance of delayed gland morphogenesis trait from Gosspium bickii into upland cotton germplasm. Chin Sci Bull.

[28] Afifi A, Bary AA, Kamel SA, Heikal I (1966). Bahtim 110, a new strain of Egyptian cotton free from gossypol. Empire Cotton Growing Rev.

[29] Carvalho LPD, Vieira RM (2000). Expression of the Gossypium barbadense Gl_2_^e^ gene in Gossypium hirsutum annual cotton. Rev De Oleaginosas Fibrosas.

[30] Dong C, Ding Y, Guo W, Zhang T (2007). Fine mapping of the dominant glandless Gene Gl_2_^e^in Sea-island cotton (Gossypium barbadense L.). Chin Sci Bull.

[31] Tang CM, Min LF, Pan JJ, Jin SY (1996). Genetic analysis for Hai1 strain of glandless cotton (G. barbadence L.): interaction between Gl_2_^e^ and Gl1. Cotton Sci Cotton Sci Sin.

[32] Cheng Hl LCR, Yu JZ, Zou CS, Zhang YP, Wang QL (2016). Fine mapping and candidate gene analysis of the dominant glandless gene Gl2e in cotton (Gossypium spp.). Theor Appl Genet.

[33] Ma D, Hu Y, Yang CQ, Liu BL, Fang L, Wan Q (2016). Genetic basis for glandular trichome formation in cotton. Nat Commun.

[34] Janga MR, Pandeya D, Campbell LM, Konganti K, Villafuerte ST, Puckhaber L (2019). Genes regulating gland development in the cotton plant. Plant Biotechnol J.

[35] Gao W, Xu FC, Lu L, Li Y, Zhang JL, Chong L (2020). The gland localized CGP1 controls gland pigmentation and gossypol accumulation in cotton. Plant Biotechnology J.

[36] Cai Y, Mo JC, Zeng Y, Ren WW, Xu Y, Wang SH (2003). Cloning of cDNAs associated with the development of pigment gland of Gossypium by suppression subtractive hybridization. J Beijing Forestry Univ.

[37] Cai YF, Cai XY, Wang QL, Wang P, Zhang Y, Cai CW, et al. Genome sequencing of the Australian wild diploid species Gossypium australe highlights disease resistance and delayed gland morphogenesis. Plant Biotechnology J. 2019:1–15. 10.1111/pbi.13249. 10.1111/pbi.13249 PMC7004908 31479566

[38] Chang PA, Li B, Ni XM, Xie YF, Cai YF (2007). Molecular cloning and expression analysis of a RanBP2 zinc finger protein gene in upland cotton (Gossypium hirsutum L.). Colloids Surfaces B: Biointerfaces.

[39] Xu YH, Wang JW, Wang S, Wang JY, Chen XY (2004). Characterization of GaWRKY1, a cotton transcription factor thatregulates the sesquiterpene synthase gene (+)-δ-cadinene synthase-A. Plant Physiol.

[40] Hong GJ, Xue XY, Mao YB, Wang LJ, Chen XY (2012). Arabidopsis MYC2 interacts with DELLA proteins in regulating sesquiterpene synthase gene expression. Plant Cell.

[41] Xie YF, Wang BC, Li B, Cai YF, Xie L, Xia YX (2007). Construction of cDNA library of cotton mutant (Xiangmian-18) library during gland forming stage. Colloids Surf B Biointerfaces.

[42] Liu WZ, Zhou YF, Wang X, Jiao ZJ (2010). Programmed cell death during pigment gland formation in Gossypium hirsutum leaves. Plant Biol.

[43] Rajhi I, Yamauchi T, Takahashi H, Nishiuchi S, Shiono K, Watanabe R (2011). Identification of genes expressed in maize root cortical cells during lysigenous aerenchyma formation using laser microdissection and microarray analyses. New Phytol.

[44] Takahashi H, Yamauchi T, Colmer TD, Nakazono M (2014). Aerenchyma formation in plants. Plant Cell Monogr.

[45] Takahashi H, Yamauchi T, Rajhi I, Nishizawa NK, Nakazono M (2015). Transcript profiles in cortical cells of maize primary root during ethylene-induced lysigenous aerenchyma formation under aerobic conditions. Ann Bot.

[46] Müller M, Munné-Bosch S (2015). Ethylene Response Factors: A Key Regulatory Hub in Hormone and Stress Signaling. Plant Physiol.

[47] Huang PY, Catinot J, Zimmerli L (2016). Ethylene response factors in Arabidopsis immunity. J Exp Bot.

[48] Rotenberg D, Thompson TS, German TL, Willis DK (2006). Methods for effective real-time RT-PCR analysis of virus-induced gene silencing. J Virol Methods.

[49] Xiong L, Schumaker KS, Zhu JK (2002). Cell signaling during cold, drought, and salt stress. Plant Cell (Suppl).

[50] Shinshi H, Usami S, Ohme-Takagi M (1995). Identification of an ethylene-responsive region in the promoter of a tobacco class I chitinase gene. Plant Mol Biol.

[51] Sessa G, Meller Y, Fluhr R (1995). A GCC element and a G-box motif participate in ethylene-induced expression of the PRB-1b gene. Plant Mol Biol.

[52] Sessa G, Meller Y, Fluhr R. A GCC element and a G-box motif participate in ethylene-induced expression of the PRB-1b gene. Plant Mol Biol (Netherlands ). 1995;28:145–53. 10.1007/BF00042046 7787179

[53] Yuan YL, Chen YH, Tang CM, Jing SR, Liu SL, Pan JJ (2000). Effects of the dominant glandless gene Gl_2_^e^ on agronomic and fiber characters of upland cotton. Plant Breed.

[54] Shi JB, Wang N, Zhou H, Xu QH, Yan GT (2020). Transcriptome analyses provide insights into the homeostatic regulation of axillary buds in upland cotton (G. hirsutum L.). BMC Plant Biology.

[55] Livak KJ, Schmittgen TD (2001). Analysis of Relative Gene Expression Data Using Real-Time Quantitative PCR and the 2<sup>−ΔΔCT</sup> Method. Methods.

[56] Burch-Smith TM, Anderson JC, Martin GB, Dinesh-Kumar SP (2004). Applications and advantages of virus-induced silencing for gene function studies in plant. Plant J.

[57] Gao X, Wheeler T, Li Z, Kenerley CM, He P, Shan L (2011). Silencing GhNDR1 and GhMKK2 compromises cotton resistance to Verticillium wilt. Plant J.

[58] Zhang JX, Wang FR, Zhang CY, Zhang JH, Chen Y, Liu GD (2018). A novel VIGS method by agro inoculation of cotton seeds and application for elucidating functions of GhBI-1 in salt-stress response. Plant Cell Rep.

[59] Gao XQ, Shan LB (2013). Functional Genomic Analysis of Cotton Genes with Agrobacterium-Mediated Virus-Induced Gene Silencing. Methods Mol Biol.

[60] Dey S, Corina VA (2015). Ethylene responsive factors in the orchestration of stress responses in monocotyledonous plants. Front Plant Sci.

[61] Jiang LL, Lian XF, Fan MS (2005). Role of programmed cell death in adaptation of plant to environmental stress. Chinese Bull Life Sci.

[62] Nayak SS, Pradhan S, Sahoo D, Parida A (2020). De novo transcriptome assembly and analysis of Phragmites karka, an invasive halophyte, to study the mechanism of salinity stress tolerance. Scientific RepoRtS.

[63] Tang ZM, Fan YJ, Zhang J, Zheng CC, Chen AY, Sun YX, Guo HX, Wu JF, Li TT, Fan YP, Lian X, Guo HH, Ma XF, Chen HF, Zeng FC. Quantitative metabolome and transcriptome analysis reveals complex regulatory pathway underlying photoinduced fiber color formation in cotton. Gene. 2020. 10.1016/j.gene.2020.145180. 10.1016/j.gene.2020.145180 33002572

[64] Yi CX, Zhang J, Chan KM, Liu XK, Hong Y (2008). Quantitative real-time PCR assay to detect transgene copy number in cotton (Gossypium hirsutum). Anal Biochem.

[65] Stefan O, Grit K, Jonathan G (2008). Increased Terpenoid Accumulation in Cotton (Gossypium hirsutum) Foliage is a General Wound Response. J Chem Ecol.

[66] Stipanovic RD, Altman DW, Begin DL (1988). GreenblattGA, Benedict JH. Terpenoid aldehydes in Upland cottons: analysis by aniline and HPLC methods. J Agric Food Chem.

