# Supplementary material for: Molecular cloning and characterization of GhERF105, a gene contributing to the regulation of gland formation in upland cotton (Gossypium hirsutum L.)
Source: BMC Plant Biol. 2021 Feb 18;21:102. doi: 10.1186/s12870-021-02846-5 (PMC7893949; doi:10.1186/s12870-021-02846-5)

Fig.S1

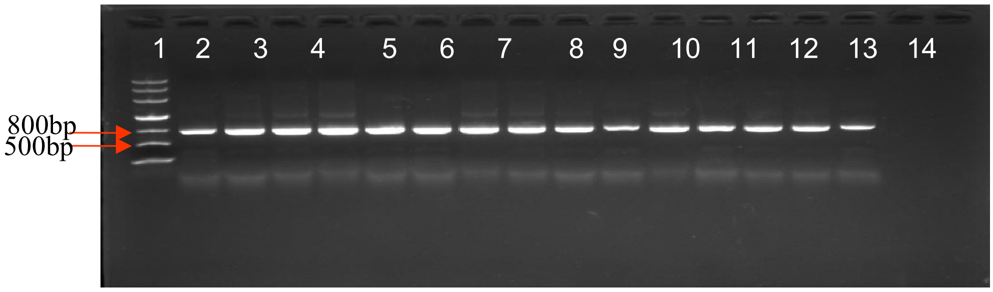

Fig.S2

MACSDETSAQLELIRQHLFTDFASMETPPPFYQLSNSESSHVYSPTRLKQSSLSQRRPSINV

MIPPTSFNISPNPVPDSAAVAVESYEKS**HYRGVRRRPWGKFAAEIRDPNKKGARVWLG**

TFDTAIEAAKAYDRAAFKLRGSKAILNFPHEAGKSNFSESGKRRRSVEEGEGEKRVSLL

ECKAGRREEEVTVAECGTPSNQTGIGDNEDINEIFGGPLLSPLSSFPWFGYSRLEVM*

Fig.S3

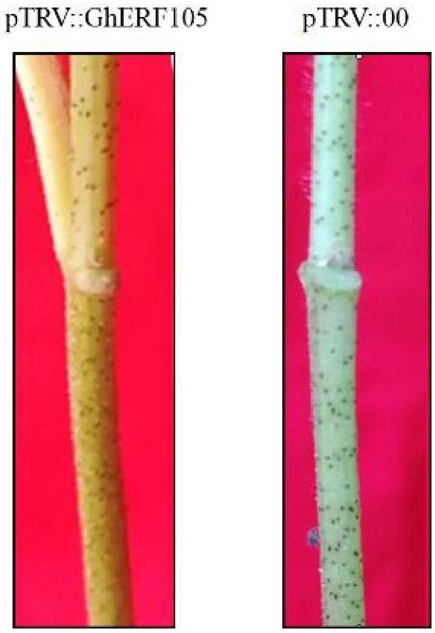

Fig.S4

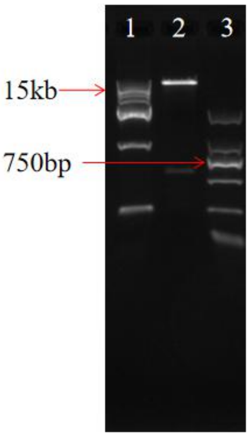

Original version (right)

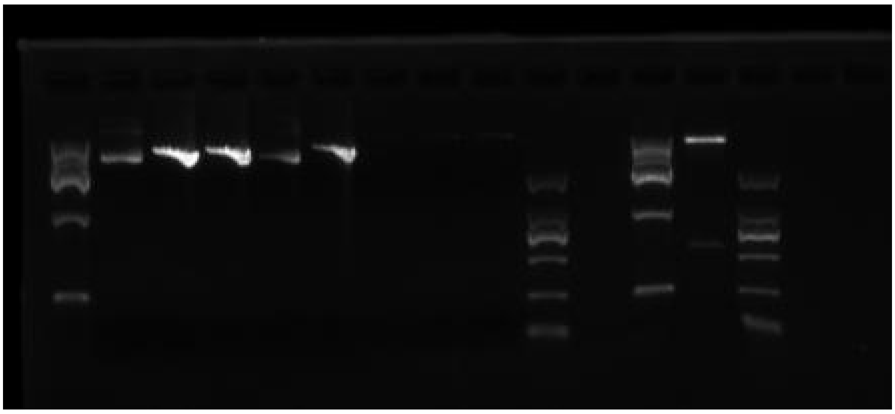

Supplement: Supplementary file 2 — Additional file 2 Fig. S1 Amplification of the full-length cDNA of GhERF105. 1: DNA marker; 2–3: the full-length cDNA of GhERF105. Fig. S2 Nucleotide and amino acid sequences of GhERF105. Symbol ‘*’ indicates the amino acid encoded by TGA, The conserved ERF domain is in black bold. Fig. S3 The phenotypes of stem inoculated with pTRV::GhERF105 and empty vector (pTRV::00). Fig. S4 The double digestion by XbaI and SmaI of result of pBI121-GhERF105-GFP construction. 1: DNA marker (DM15000), 2 double digestion by endonuclease of recombinant vector. 3 DNA marker (DM2000). [file 12870_2021_2846_MOESM2_ESM.pdf]
